# Supplementary material for: Bioinspired rotary flight of light-driven composite films
Source: Nat Commun. 2023 Aug 21;14:5070. doi: 10.1038/s41467-023-40827-4 (PMC10442326; doi:10.1038/s41467-023-40827-4)
Supplement: Supplementary file 1 — Supplementary Information [file 41467_2023_40827_MOESM1_ESM.pdf]

Supplementary Information

Bioinspired rotary flight of light-driven composite films

Dan Wang,[1,2#] Zhaomin Chen,[1#] Mingtong Li,[3#] Zhen Hou,[1] Changsong Zhan,[1] Qijun Zheng,[4] Dalei Wang,[1] Xin Wang,[1] Mengjiao Cheng,[2] Wenqi Hu,[3] Bin Dong,[1*] Feng Shi[2*] and Metin Sitti [3,5,6*]

[1]Institute of Functional Nano & Soft Materials (FUNSOM), Jiangsu Key Laboratory for Carbon-Based Functional Materials & Devices, State and Local Joint Engineering Laboratory for Novel Functional Polymeric Materials & Joint International Research Laboratory of Carbon-Based Functional Materials and Devices, Soochow University, Suzhou, Jiangsu 215123, China

[2]State Key Laboratory of Chemical Resource Engineering, Beijing Laboratory of Biomedical Materials & Beijing Advanced Innovation Center for Soft Matter Science and Engineering, Beijing University of Chemical Technology, Beijing, 100029, China

[3]Physical Intelligence Department, Max Planck Institute for Intelligent Systems, 70569 Stuttgart, Germany

[4]Department of Chemical Engineering, Monash University, Clayton, Vic 3800, Australia

[5]Institute for Biomedical Engineering, ETH Zürich, 8092 Zürich, Switzerland

[6]School of Medicine and College of Engineering, Koç University, 34450 Istanbul, Turkey

[#]These authors contributed equally to this work.

*Correspondence to: sitti@is.mpg.de, shi@mail.buct.edu.cn, bdong@suda.edu.cn

Table of content

Section 1. Supplementary Tables

Section 2. Supplementary Figures

Supplementary Movies 1-12

Section 1. Supplementary Tables

Supplementary Table 1. Strategies that have been developed to improve the response of the photoactuators.

Photoactuator material	Shape	Response	Mechanism	Ref.
Liquid crystal gels (LCGs)	Strip	>1 s	Phase transition (in water)	1
SWNT/VO_2 based actuators	Cantilever	3.3 ms	Phase transition	2
Iron oxide nanoparticle and poly(sodium acrylate) hydrogel actuator	Sphere	800 ms	Synergetic interactions	3
Ink/polyethylene terephthalate (PET)/acrylic actuator	Strip	360 ms	Synergetic interactions	4

In order to improve the response of the photoacutators, researchers have developed several strategies. Among others, the utilization of the low phase temperature materials has been demonstrated to be an effective way to improve the response. For example, the liquid crystal gel exhibits a low phase transition temperature (57 °C) so that it shows almost 30 times higher photothermal response than that of the pristine liquid crystal networks.[1] In addition, the response of the phase-change material (VO_2 with the low phase transition temperature of 68 °C) based

photoactuator could be further improved by 36 % when introducing materials with increased light absorbing ability and decreased thermal conductivity.[2] On the other hand, the synergetic interactions are frequently exploited to further enhance the response. For instance, the elasticity of the hydrogel and the photothermally induced phase transition could be combined, leading to the fast response (only 800 ms) of the hydrogel in air.[3] Furthermore, the synergistic effect between photothermal expansion and water desorption has been utilized to increase the response of the trilayered photoactuator to 360 ms.[4]

Supplementary Table 2. Comparison of the rotational speeds of photoactuators with different shapes and driving mechanisms.

Photoactuator material	Shape	Rotation speed (rpm)	Mechanism	Ref.
Graphene/agar/silk fibroin composite	Airscrew	7200	Jet propulsion	This work
Carbonized polydimethylsiloxane (PDMS)	Microgear	94.2	Marangoni effect	5
Carbon/SU-8 photoresist	Microgear	300	Marangoni effect	6
Pyrolytic graphite	Disk	200	Magnetic susceptibility change	7
Ink/PET/acrylic adhesive	Light mill	19	Wing-flapping	4

Section 2. Supplementary Figures.

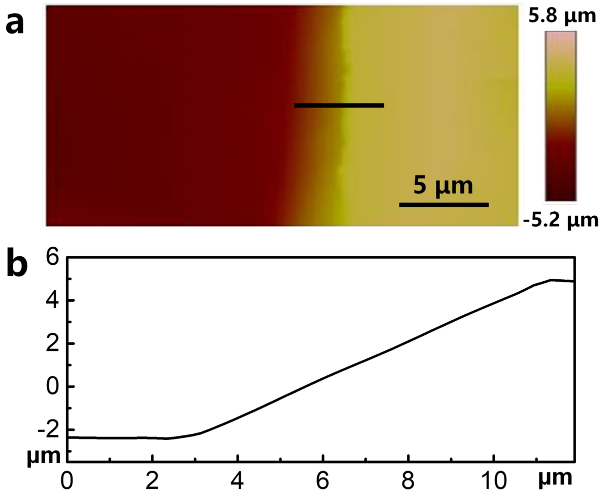

Supplementary Fig. 1. (a) The atomic force microscopy (AFM) image showing the edge of the microchannel on the surface of the graphene/agar/silk fibroin photoactuator film. The corresponding section analysis of (a) is shown in (b).

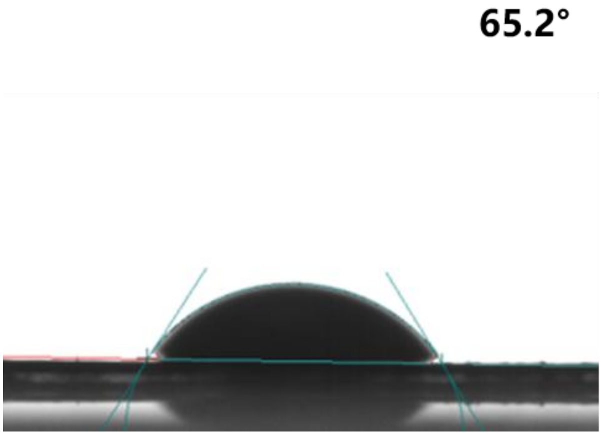

Supplementary Fig. 2. The water contact angle on the graphene/agar/silk fibroin photoactuator film.

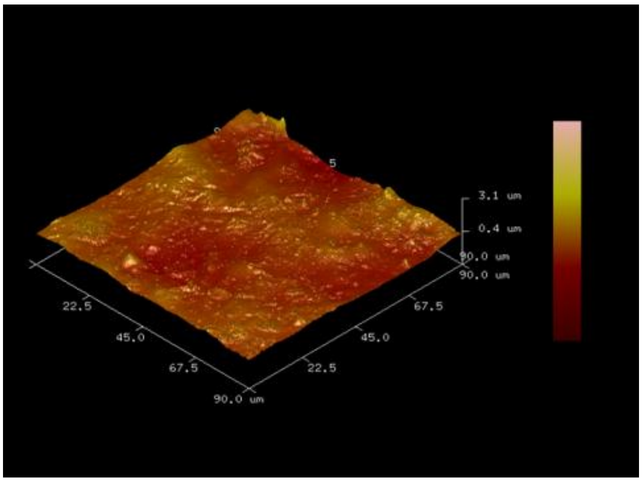

Supplementary Fig. 3. The AFM image showing the 3D surface morphology of the graphene/agar/silk fibroin photoactuator film.

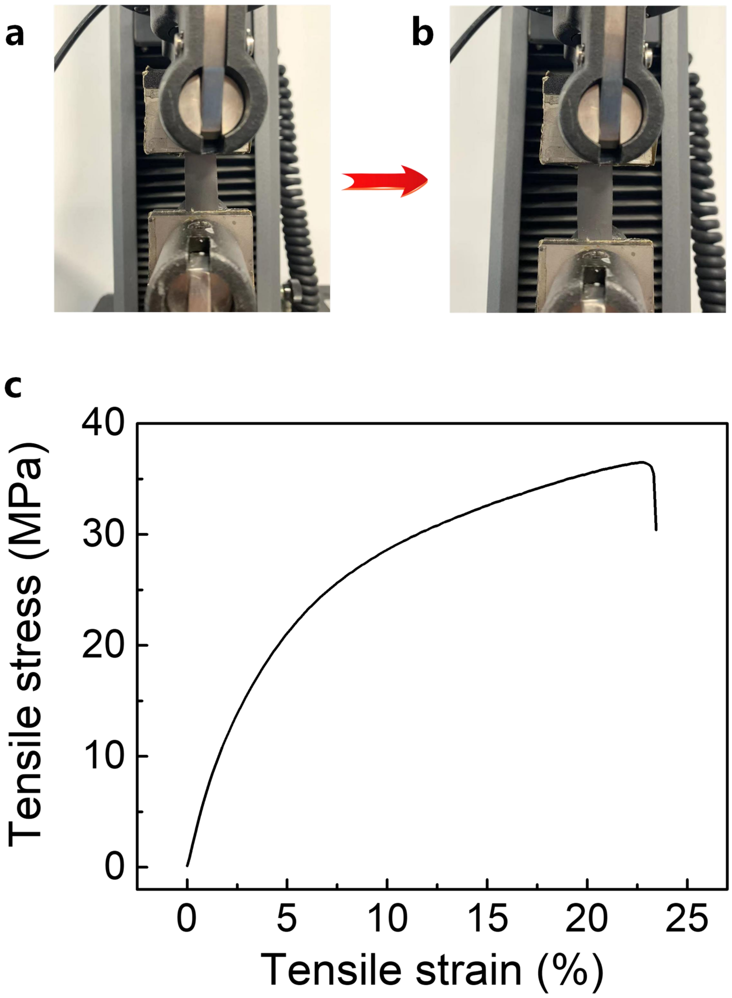

Supplementary Fig. 4. A strip-shaped graphene/agar/silk fibroin photoactuator film (a) before and (b) after stretching. (c) Tensile stress-strain curve of the graphene/agar/silk fibroin photoactuator film.

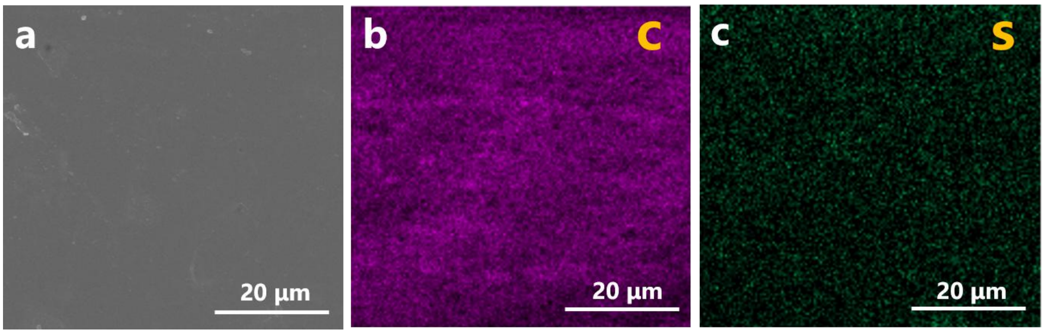

Supplementary Fig. 5. (a) Scanning electron microscopy (SEM) image showing the surface morphology of the graphene/agar/silk fibroin photoactuator film. Energy-dispersive X-ray (EDX) analysis of the graphene/agar/silk fibroin photoactuator film showing the homogeneous distribution of (b) carbon and (c) sulfur elements.

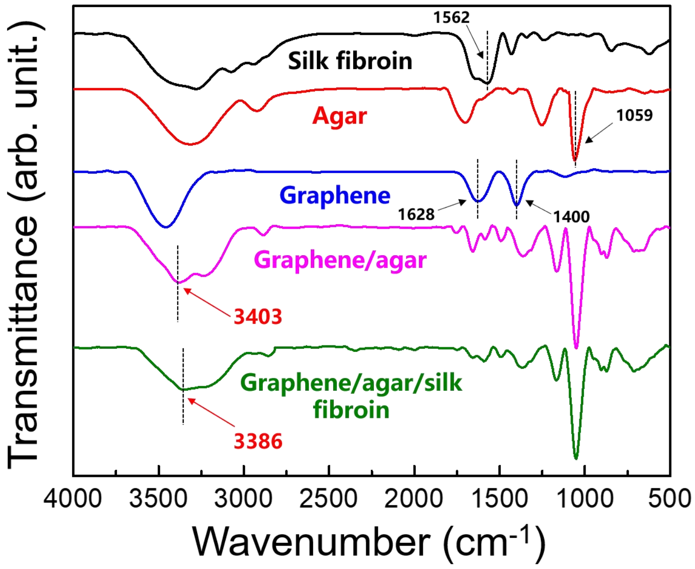

Supplementary Fig. 6. Fourier transform infrared spectroscopy (FTIR) spectra of silk fibroin (black curve), agar (red curve), graphene (blue curve), graphene/agar film (purple curve) and the graphene/agar/silk fibroin photoactuator film (green curve).

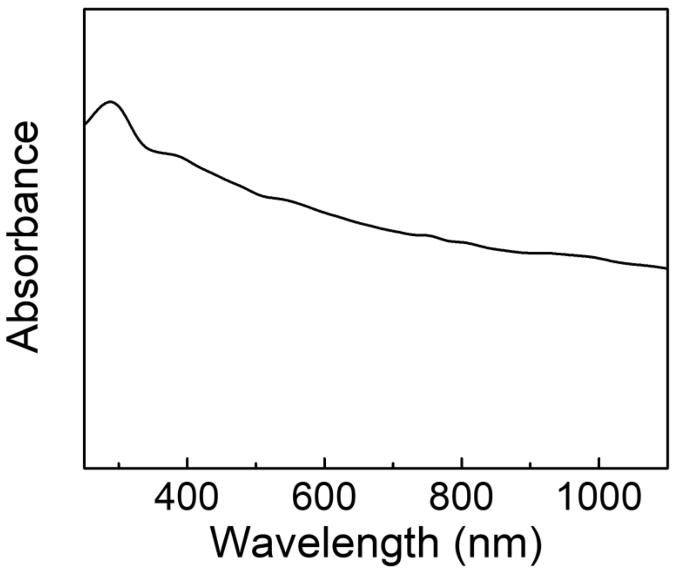

Supplementary Fig. 7. The UV-Vis-NIR spectrum of the graphene/agar/silk fibroin solution.

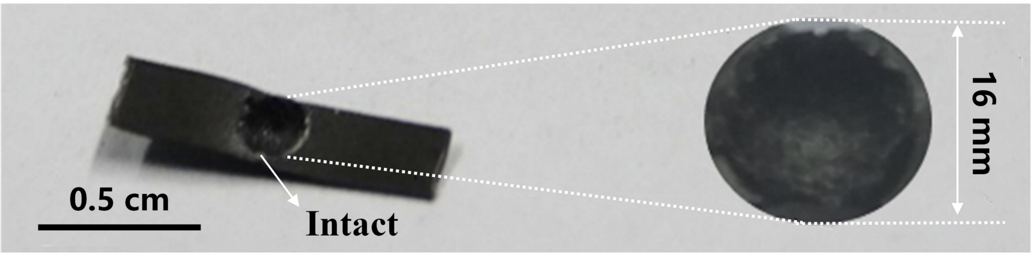

Supplementary Fig. 8. CCD images showing the sectioned protrusion which indicate the hollow interior structure. Note that the protrusion only expands to one side of the film and the opposite side remains intact.

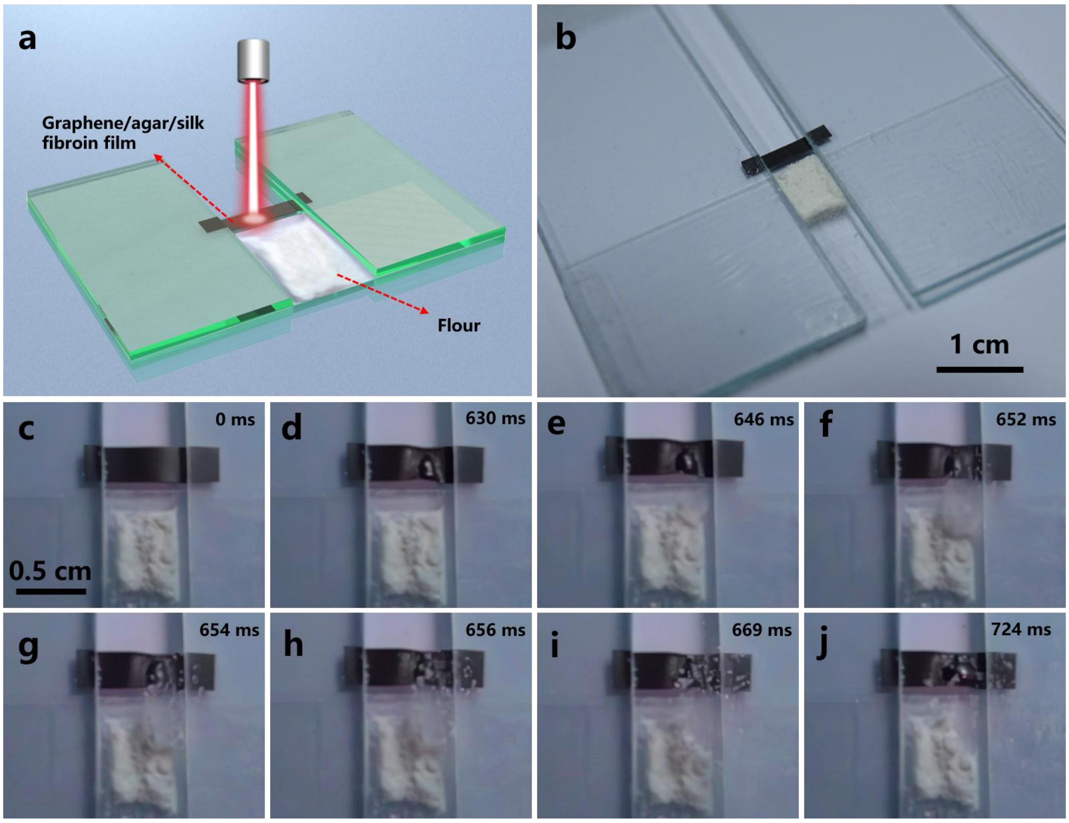

Supplementary Fig. 9. (a) Schematic illustration indicating the experimental setup for the verification of the jet propulsion. Time lapse images obtained from Supplementary Movie 3 showing the graphene/agar/silk fibroin photoactuator film (b,c) before and (d-j) after light actuation.

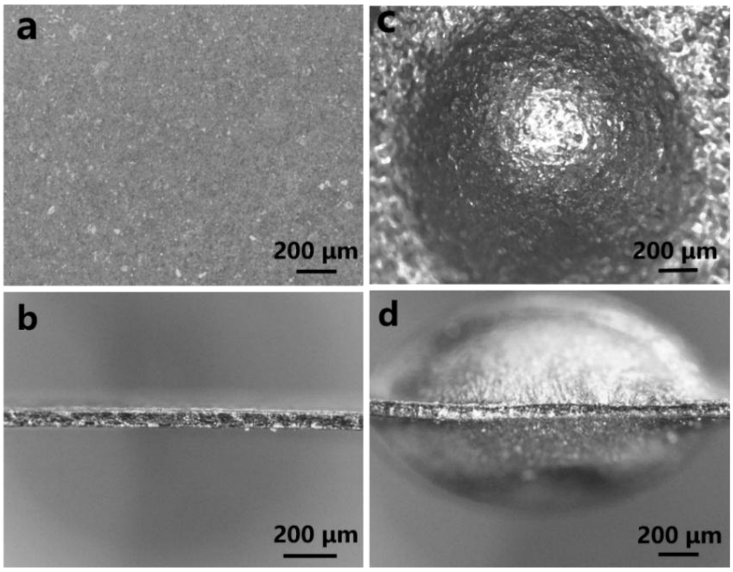

Supplementary Fig. 10. Microscopic images showing the structure of the graphene/agar/silk fibroin photoactuator film (a-b) before and (c-d) after light actuation: (a,c) top view and (b,d) cross-sectional view. There are negligible changes at the film edge where the jet propulsion occurs.

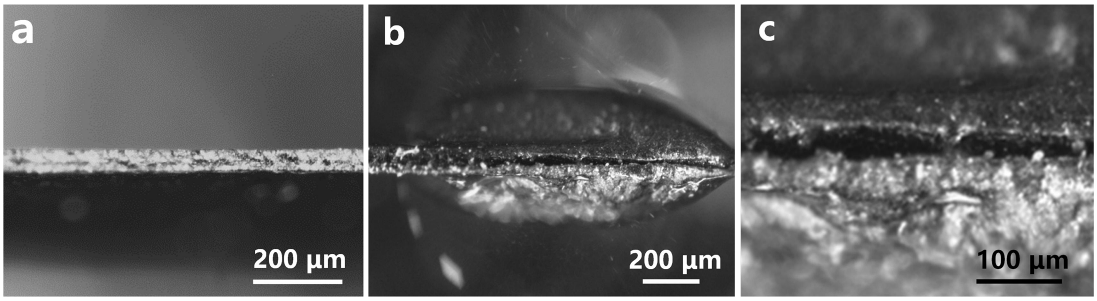

Supplementary Fig. 11. Optical microscopic images showing the cross-section of the graphene/agar photoactuator film (in the absence of silk fibroin) (a) before and (b) after light actuation. (c) Enlarged microscopic image showing the split at the film edge after the jet propulsion.

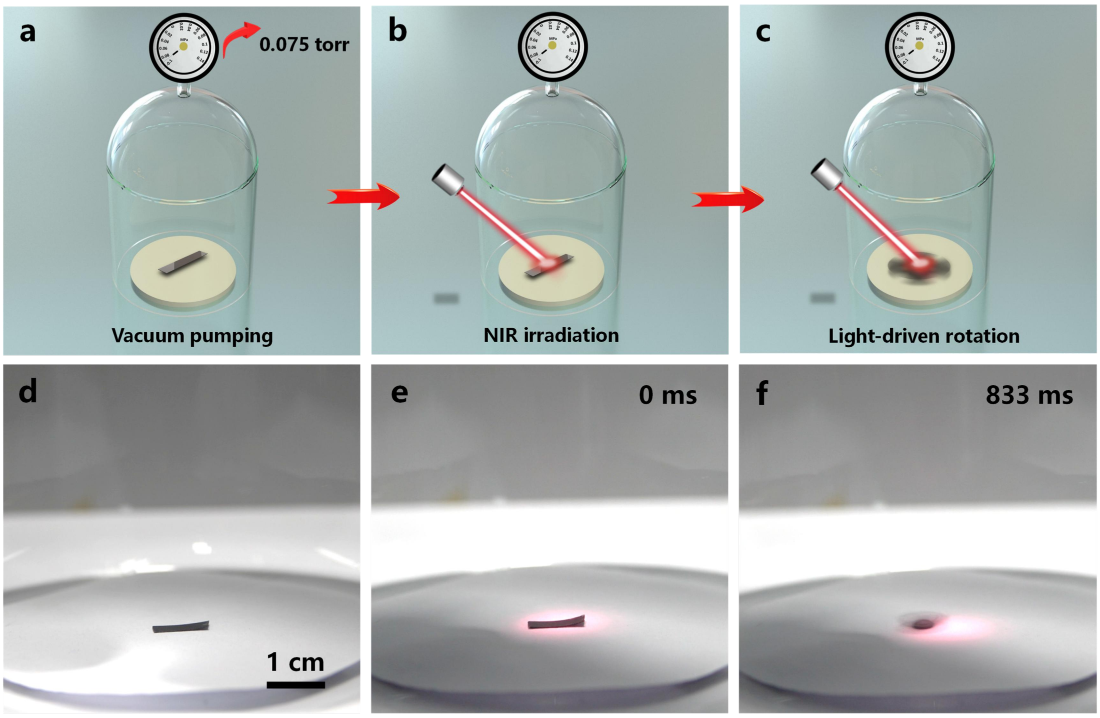

Supplementary Fig. 12. (a-c) Schematic illustration and (d-f) the corresponding time lapse images obtained from Supplementary Movie 4 indicating the light-driven rotation of the graphene/agar/silk fibroin photoactuator film in vacuum (0.075 torr).

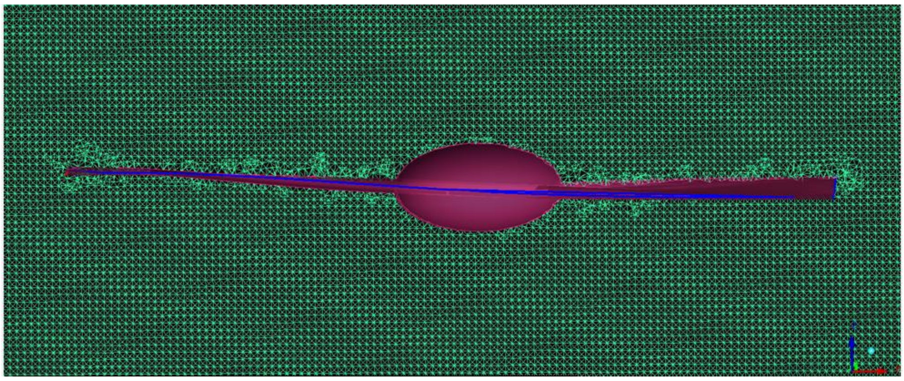

Supplementary Fig. 13. Computation mesh used for the computational fluid dynamics (CFD) simulation.

The 3D models and computational domains at different angle of attack are created using the CFD module. During this process, the whole computational domain is divided by unstructured mesh. The computational mesh of a 10 mm × 2 mm × 60 μm (length × width × thickness) sized film with an angle of attack of 15° is shown in Supplementary Fig. 13. The computational boundary (x, y and z) domains are set as 5, 2 and 10 times of the length, width and thickness of the film, respectively (the number of meshes is 5,000,000). The boundary conditions are set as follows: The inlet condition is set as the velocity inlet, the outlet condition is set as a zero-gradient pressure outlet and a uniform free incoming velocity is used. The wall boundary condition with no slip and no penetration is used for the film surface. The number of boundary mesh layers is 10, the thickness of the first layer is 0.001 times of the thickness of the film.

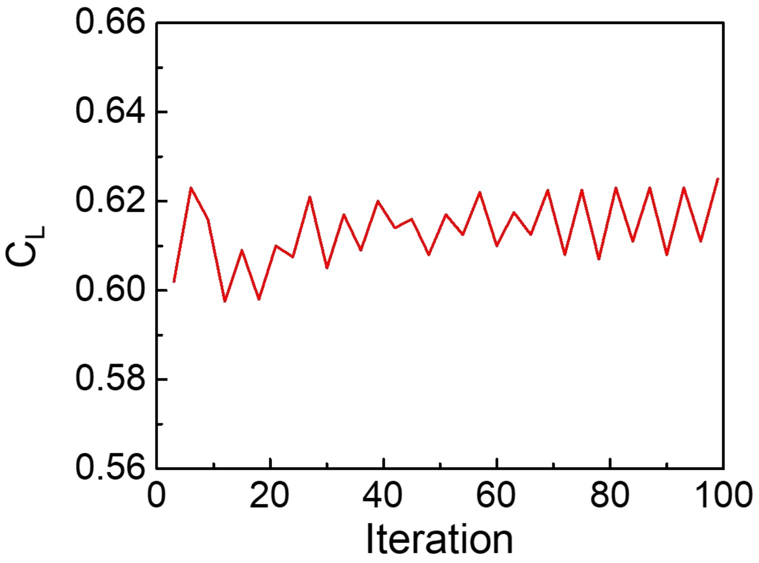

Supplementary Fig. 14. Lift coefficient (C_L) of the helicopter-like photoactuator.

The k-epsilon model in Fluent module is used, the fluid is set as air (the air flow rate is set as 10 m/s) and the characteristic area of the reference value is 0.23 cm^2. Accordingly, the average simulated value of the lift coefficient (C_L) based on the k-epsilon model is 0.61, as shown in Supplementary Fig. 14. Therefore, the lift force can be calculated to be 4.12×10^{-4} N, which is in the same order of magnitude as the lift force calculated based on Equation 1 (1.44×10^{-4} N), thus demonstrating that the rotation-induced lift force is likely to be the driving force for flight.

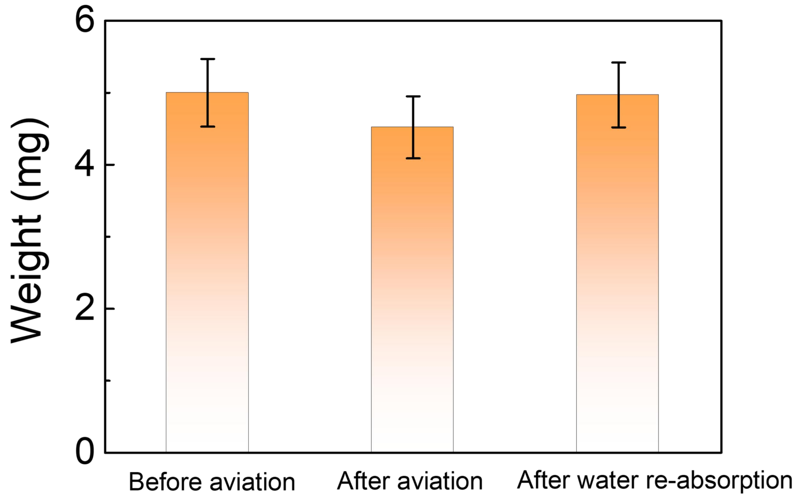

Supplementary Fig. 15. The weight variation of the graphene/agar/silk fibroin photoactuator film before light-driven aviation, after light-driven aviation and after water re-absorption. Error bars denote the standard deviation.

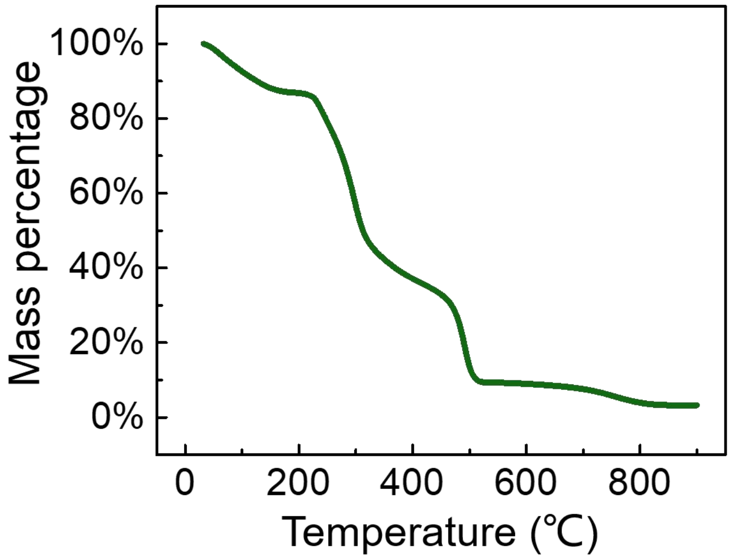

Supplementary Fig. 16. The thermogravimetric analysis (TGA) curve of the graphene/agar/silk fibroin photoactuator film.

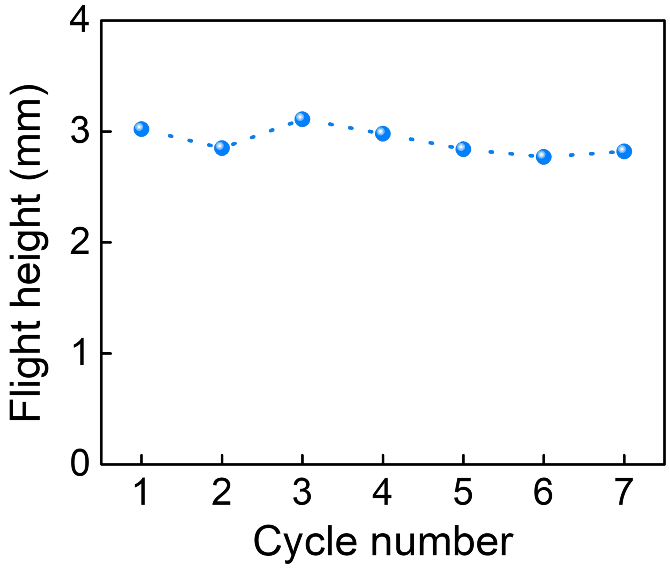

Supplementary Fig. 17. The repeatability of the light-driven flight of the helicopter-like photoactuator under 0.3 W/cm^2 repetitive light actuation.

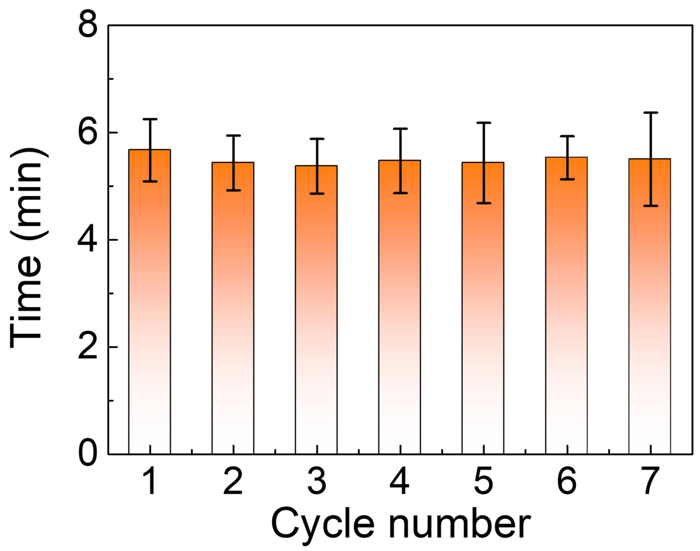

Supplementary Fig. 18. Water re-absorption time of the helicopter-like photoactuator after repetitive actuation. Error bars denote the standard deviation.

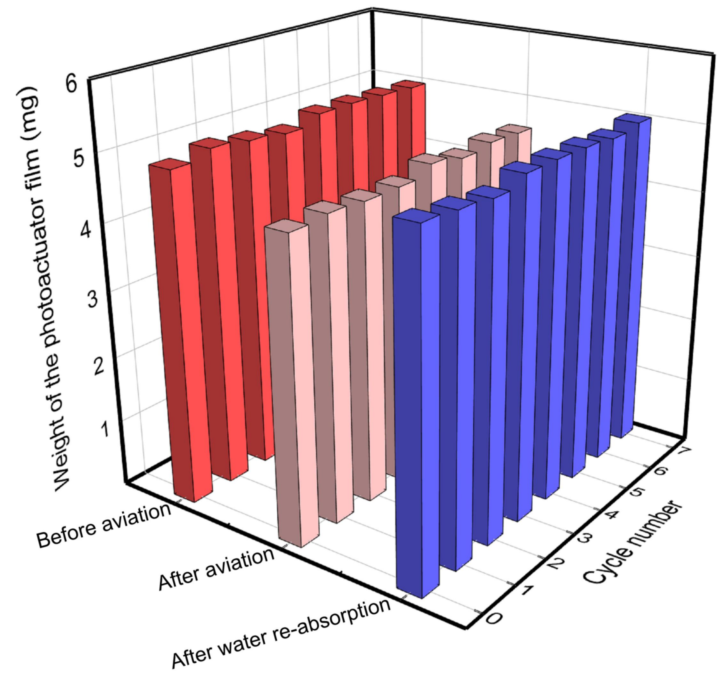

Supplementary Fig. 19. Weight variation of the photoactuator film before aviation (red), after aviation (pink) and after water re-absorption (blue) for 7 cycles.

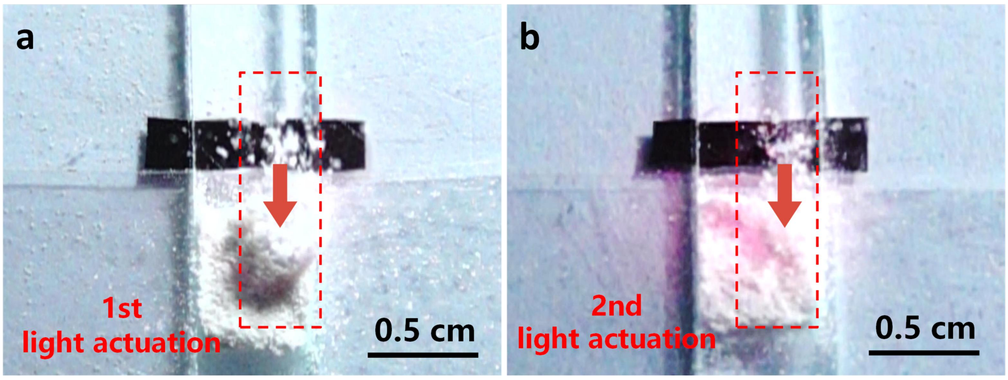

Supplementary Fig. 20. The images showing the photoactuator film after (a) first and (b) second light actuation. The generated jet propulsion can be directly visualized by the blown white powders. After light actuation, the white powders are blown away basically at the same position for the first two cycles, which indicates the jet propulsion position may be unchanged.

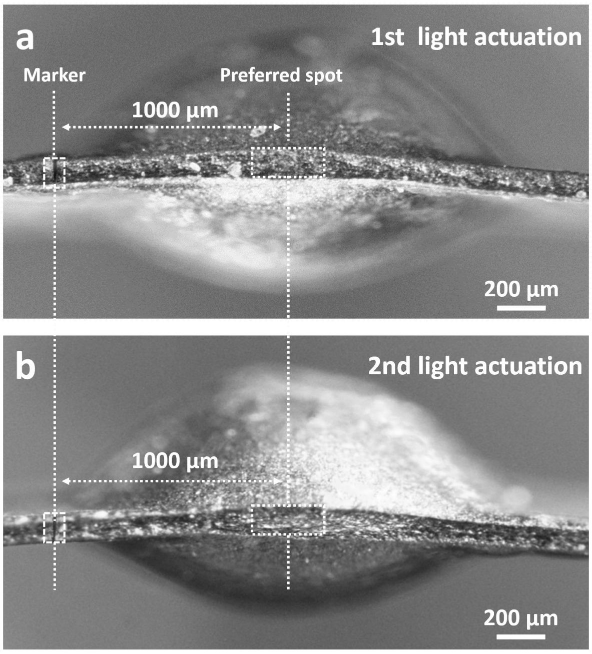

Supplementary Fig. 21. Microscopic images showing the cross-section of the film edge where the jet propulsion occurs after (a) first and (b) second light actuation. Note that there is a marker which is formed by cutting the film edge prior to the light actuation. The distance from the marker to the jet propulsion position is approximately 1 mm after the first light actuation and remains unchanged after the second cycle of light actuation.

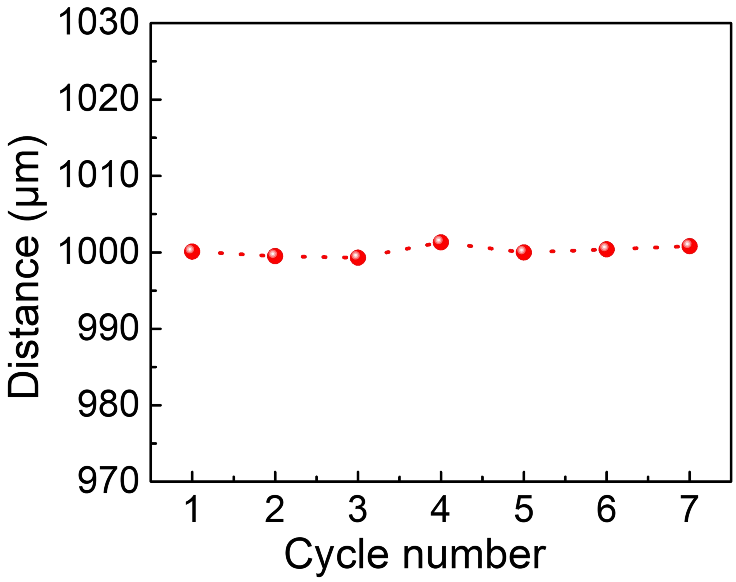

Supplementary Fig. 22. Distance from the marker to the preferred spot (i.e. the jet propulsion position) after light actuation for 7 cycles. The distance variation is negligible. This further indicates that, after the initial jetting, a preferred spot forms at the film edge, and the water vapor consistently escapes from this preferred spot during the subsequent cycles.

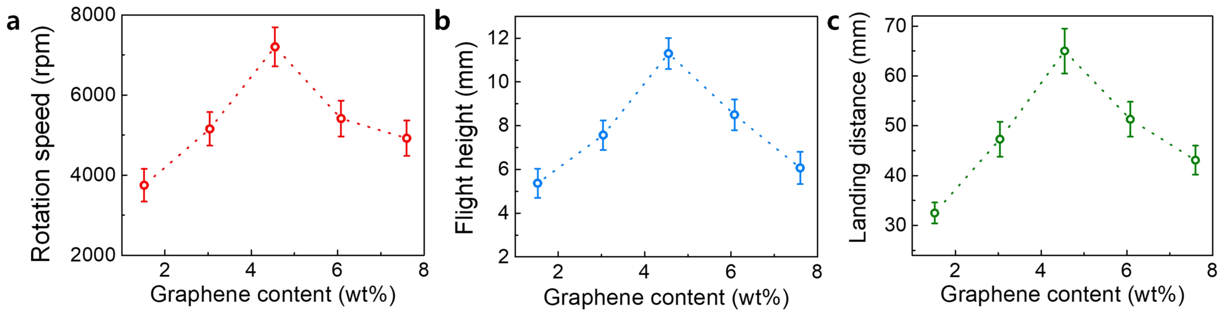

Supplementary Fig. 23. The influence of the graphene content on (a) the rotational speed, (b) the flight height and (c) the landing distance of the helicopter-like photoactuator. Error bars denote the standard deviation.

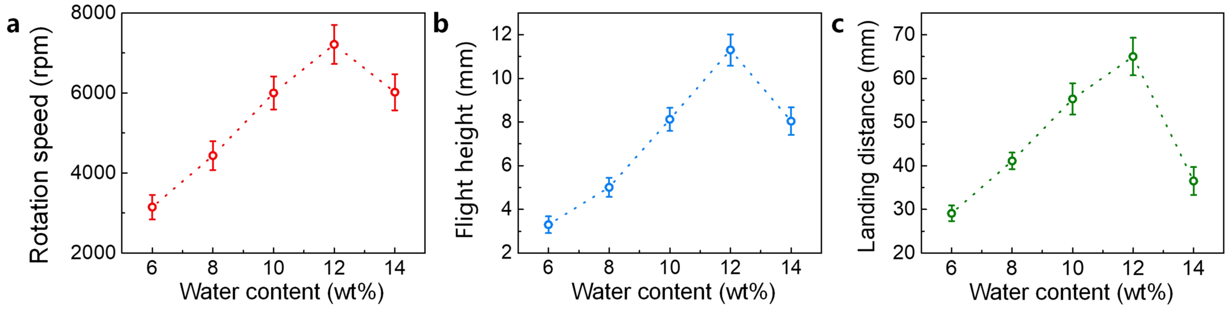

Supplementary Fig. 24. The influence of the water content on (a) the rotational speed, (b) the flight height and (c) the landing distance of the helicopter-like photoactuator. Error bars denote the standard deviation.

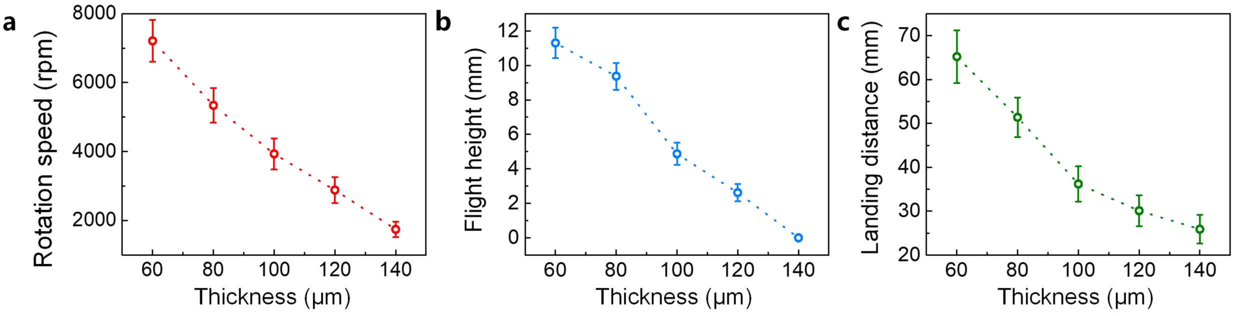

Supplementary Fig. 25. The influence of the thickness of the photoactuator film on (a) the rotational speed, (b) the flight height and (c) the landing distance of the helicopter-like photoactuator. Error bars denote the standard deviation.

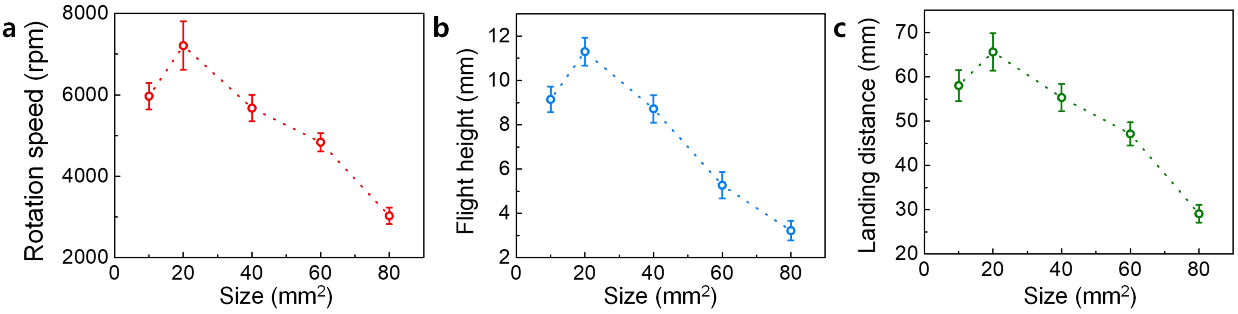

Supplementary Fig. 26. The influence of size of the photoactuator film on (a) the rotational speed, (b) the flight height and (c) the landing distance of the helicopter-like photoactuator. Error bars denote the standard deviation.

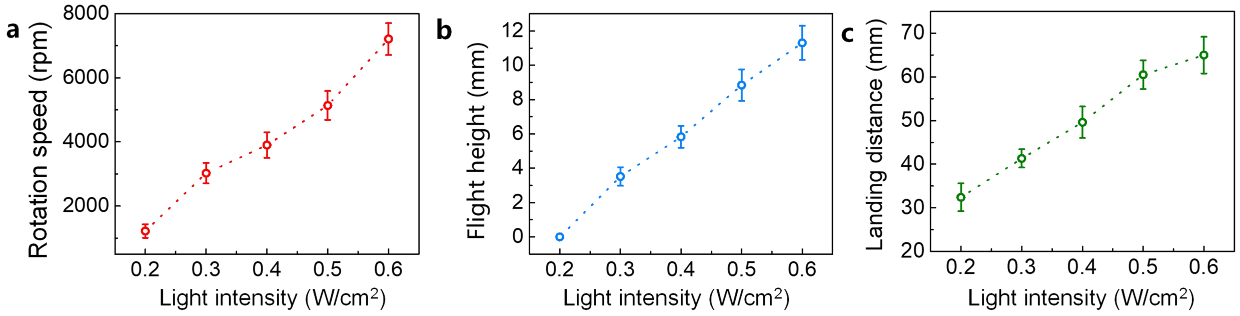

Supplementary Fig. 27. The influence of the irradiation light intensity on (a) the rotational speed, (b) the flight height and (c) the landing distance of the helicopter-like photoactuator. Error bars denote the standard deviation.

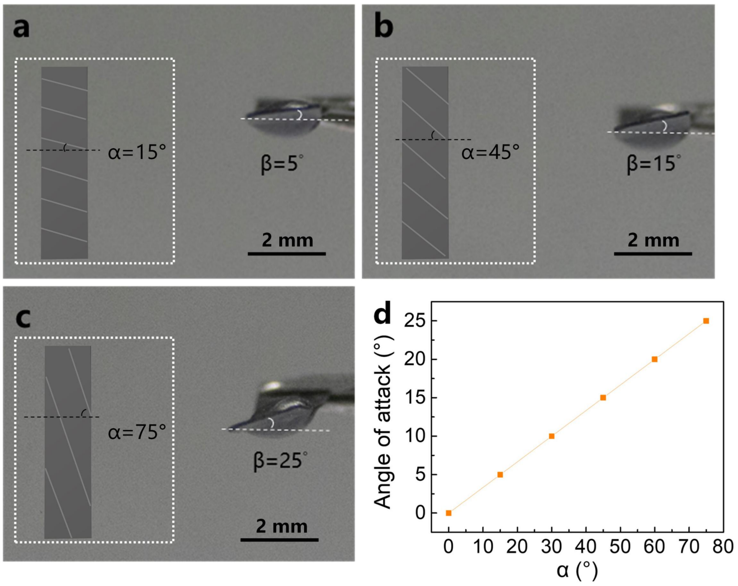

Supplementary Fig. 28. (a-c) CCD images showing the angle of attack (β) of the helicopter-like photoactuator could be controlled by controlling the alignment angle (α) of the microchannels (with respect to the short axis of the photoactuator film). The corresponding schematic illustrations are shown in the insets (dashed boxes) in (a-c). (d) Angle of attack depends on the alignment angle of the microchannels (α).

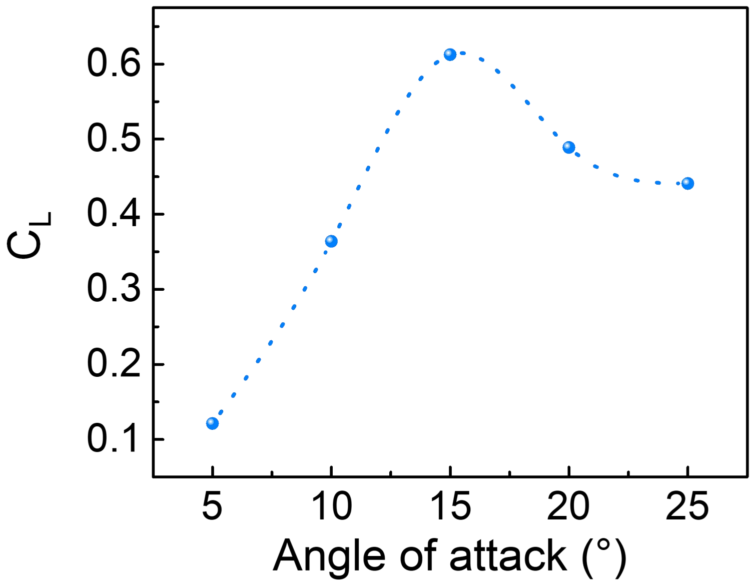

Supplementary Fig. 29. The CFD simulation showing the influence of the angle of attack on the lift coefficient (C_L). C_L first increases gradually with the increase of the angle of attack and then decreases. The optimal angle of attack is 15°, at which C_L reaches the maximum value of 0.61.

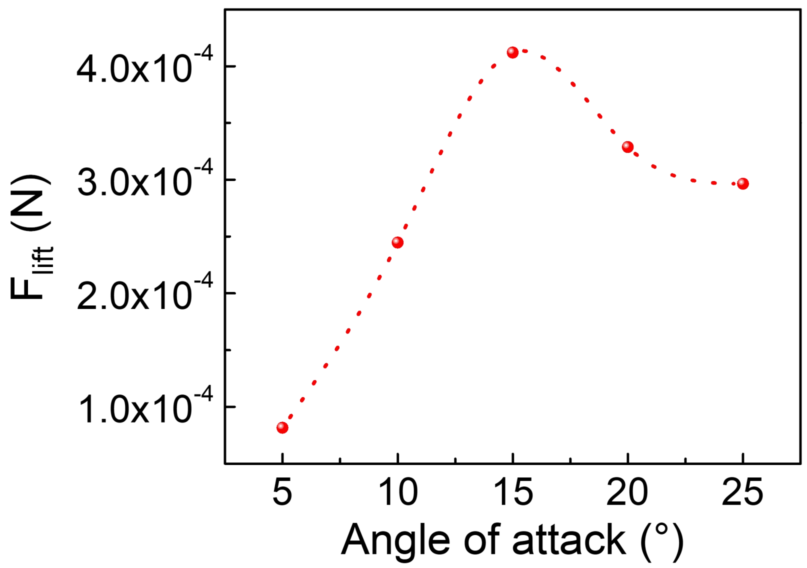

Supplementary Fig. 30. The CFD simulation showing the influence of the angle of attack on the lift force (F_{lift}).

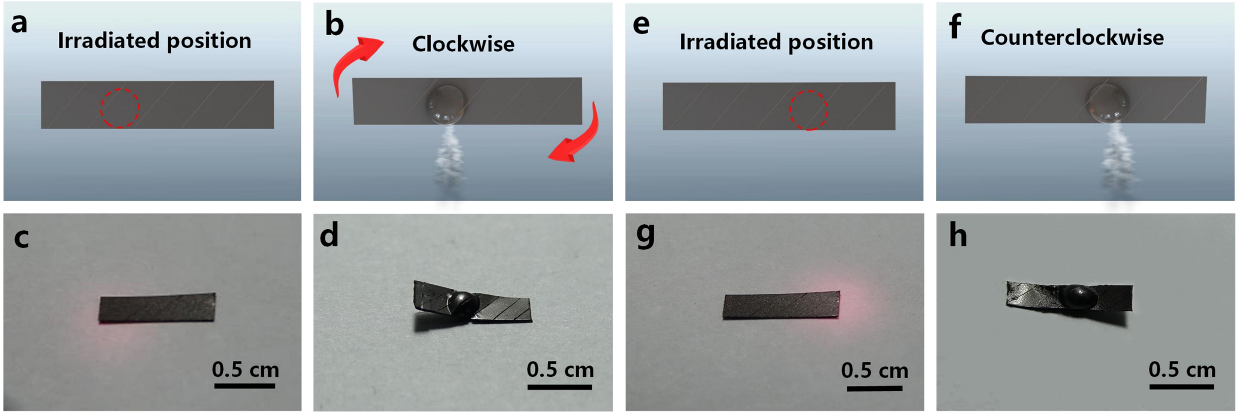

Supplementary Fig. 31. Schematic illustration and CCD images showing the controlled (a-d) clockwise and (e-h) counterclockwise rotation which is realized by controlling the position of the jet propulsion. Note that, the jet propulsion at the lower left part of the photoactuator film (a-d) induces the clockwise rotation, while that at the lower right part of the photoactuator film (e-h) leads to the counterclockwise rotation.

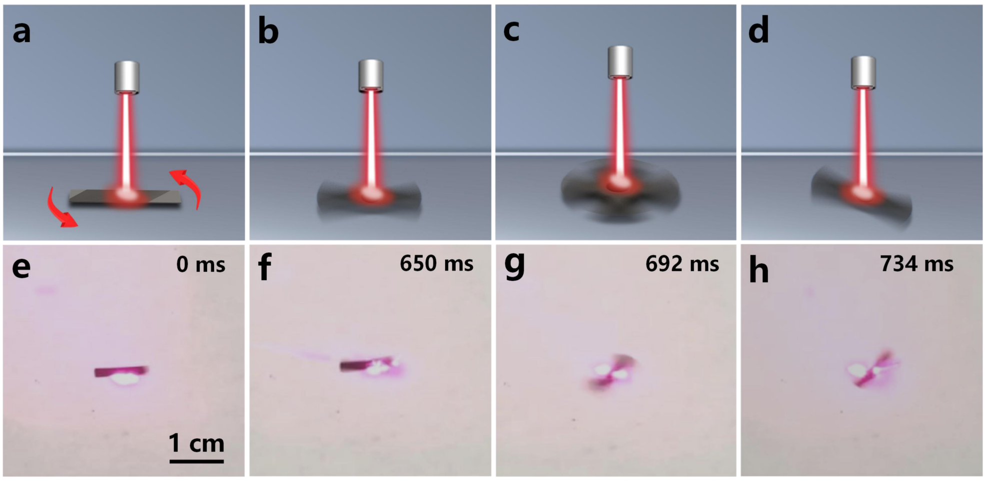

Supplementary Fig. 32. (a-d) Schematic illustration and (e-h) the corresponding time lapse images obtained from Supplementary Movie 5 showing the graphene/agar/silk fibroin photoactuator film only rotates when the angle of attack is negative.

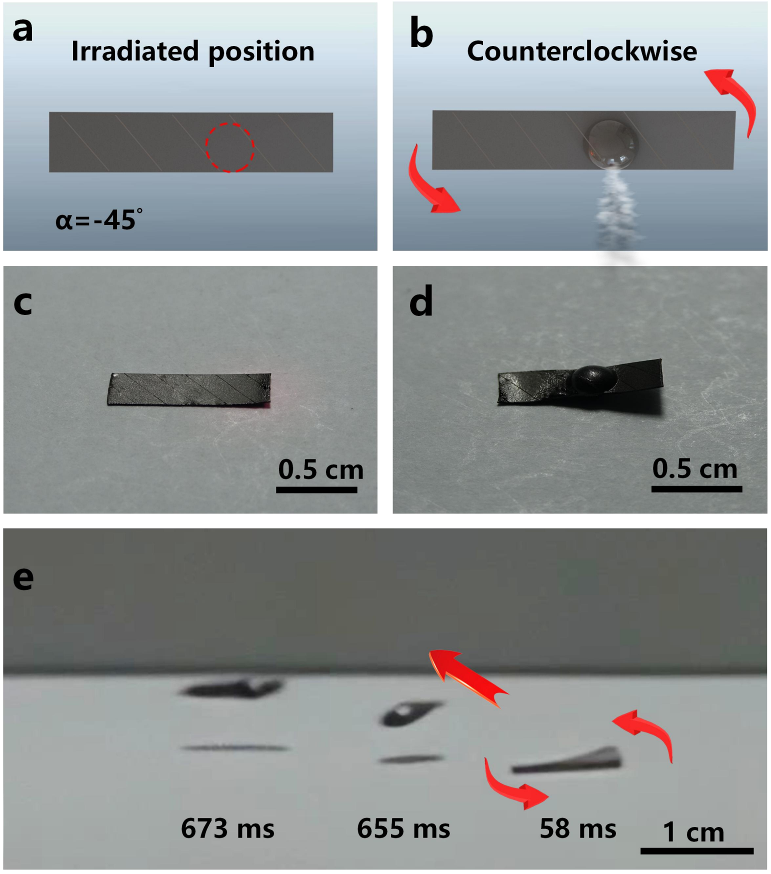

Supplementary Fig. 33. (a,b) Schematic illustration, (c,d) the CCD images and (e) the overlaid CCD images showing that, by changing the alignment angle of the microchannels (α) (from 45° in case of Supplementary Fig. 31 to -45°), the counterclockwise rotation could result in the flying motion.

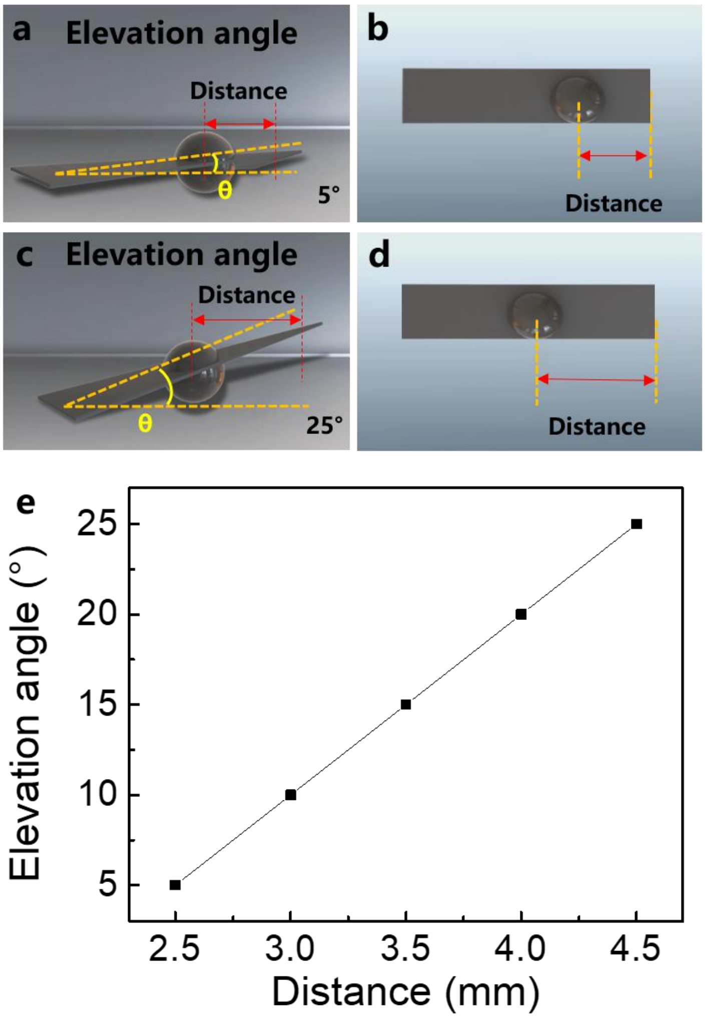

Supplementary Fig. 34. Schematic illustration showing the elevation angle (θ) of (a,b) 5° and (c,d) 25° which is adjusted by controlling the position of the protrusion. (e) Elevation angle could be controlled by controlling the distance away from the right end of the photoactuator film

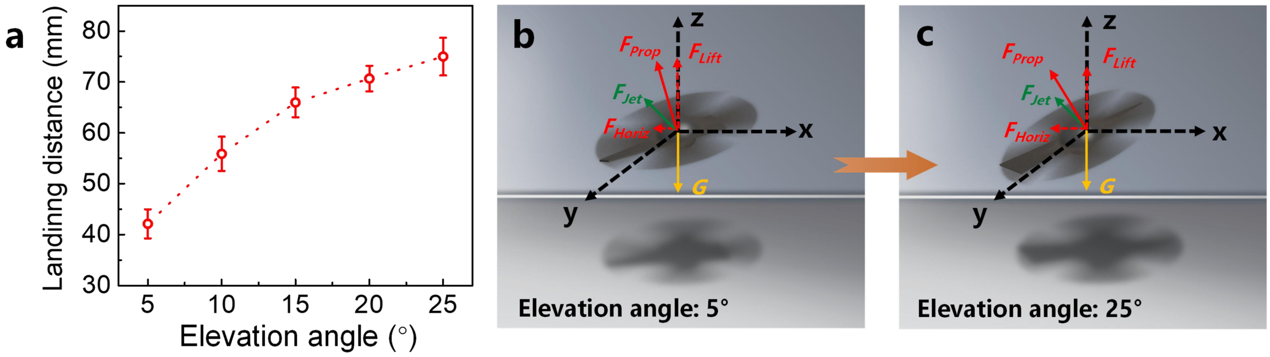

Supplementary Fig. 35. (a) The landing distance as a function of the elevation angle of the helicopter-like photoactuator. Force analysis of the helicopter-like photoactuator with elevation angle of (b) 5° and (c) 25°, respectively. F_{prop}, solid red arrow, is the propulsion force generated as a result of the fast rotation. F_{horiz}, dashed red arrow, represents the horizontal component force. F_{lift}, dashed red arrow, is the vertical component force (lift force). G, solid yellow arrow, is the gravitational force of the photoactuator. Error bars denote the standard deviation.

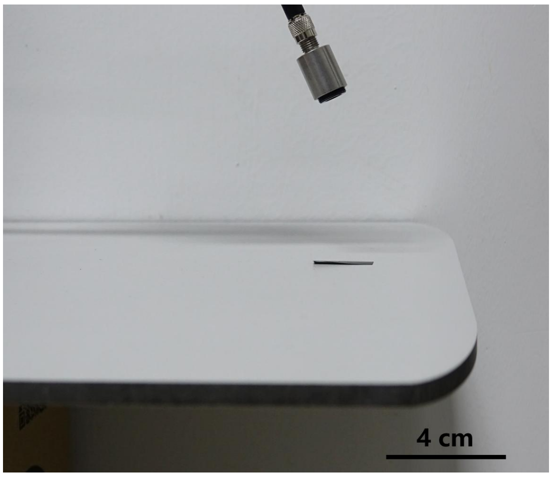

Supplementary Fig. 36. CCD image showing the experimental setup for the helicopter-like photoactuator released upon light actuation.

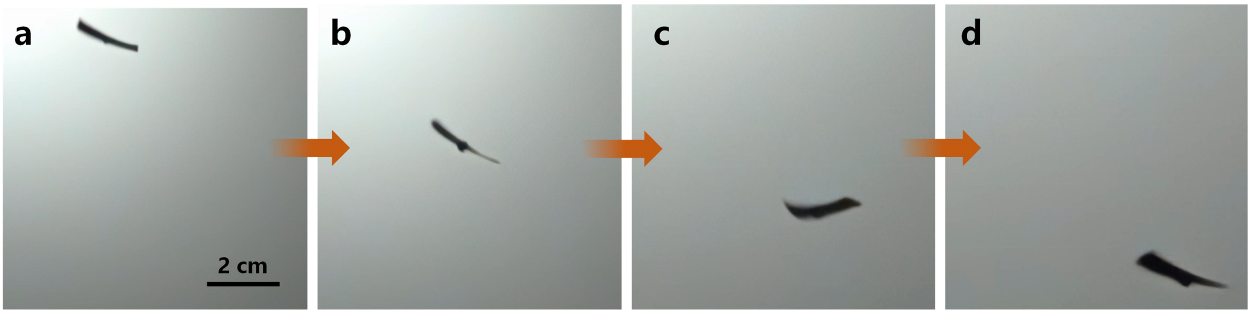

Supplementary Fig. 37. (a-d) Time lapse images within 74 ms obtained from Supplementary Movie 9 showing the apparent horizontal displacement of the graphene/agar/silk fibroin photoactuator film during the rotary-falling process in the presence of a crosswind (the wind speed is 3 m/s).

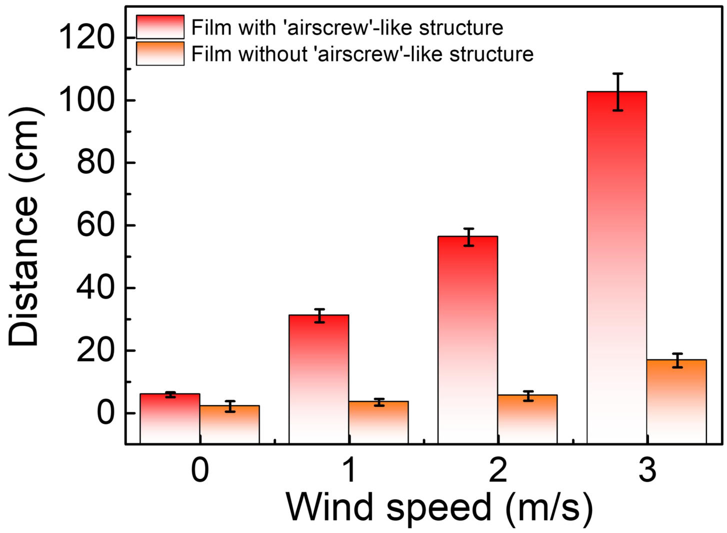

Supplementary Fig. 38. The wind-dispersal distance of the composite film with (red column) and without (orange column) the airscrew-like structure. Error bars denote the standard deviation.

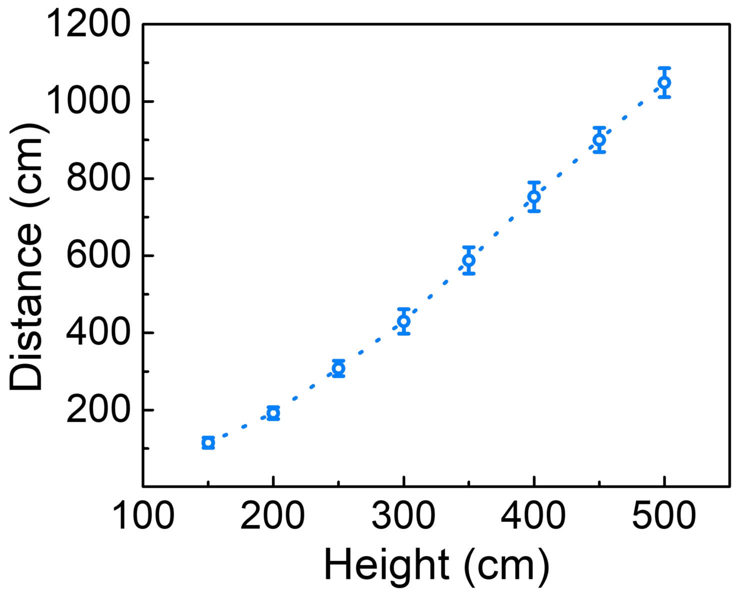

Supplementary Fig. 39. Wind-dispersal distance of the photoactuator released from different height. Error bars denote the standard deviation.

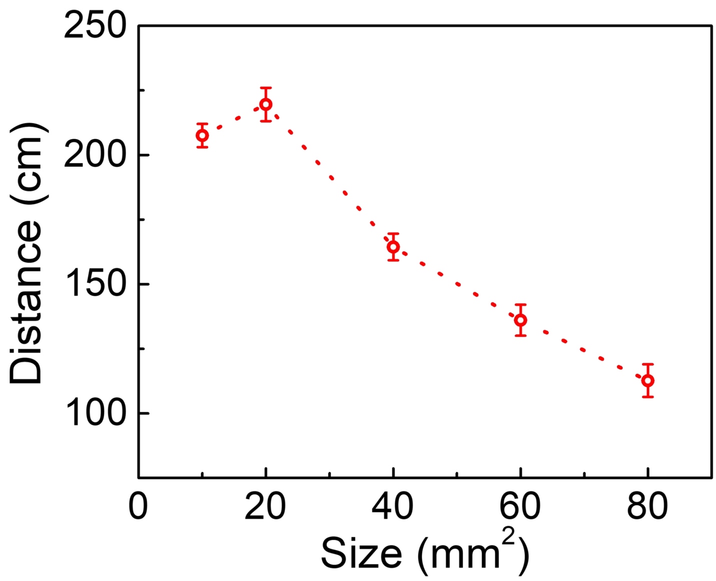

Supplementary Fig. 40. The effect of the size of the photoactuator on its wind-dispersal distance (the release height and the wind speed are 1.5 m and 3 m/s, respectively). Error bars denote the standard deviation.

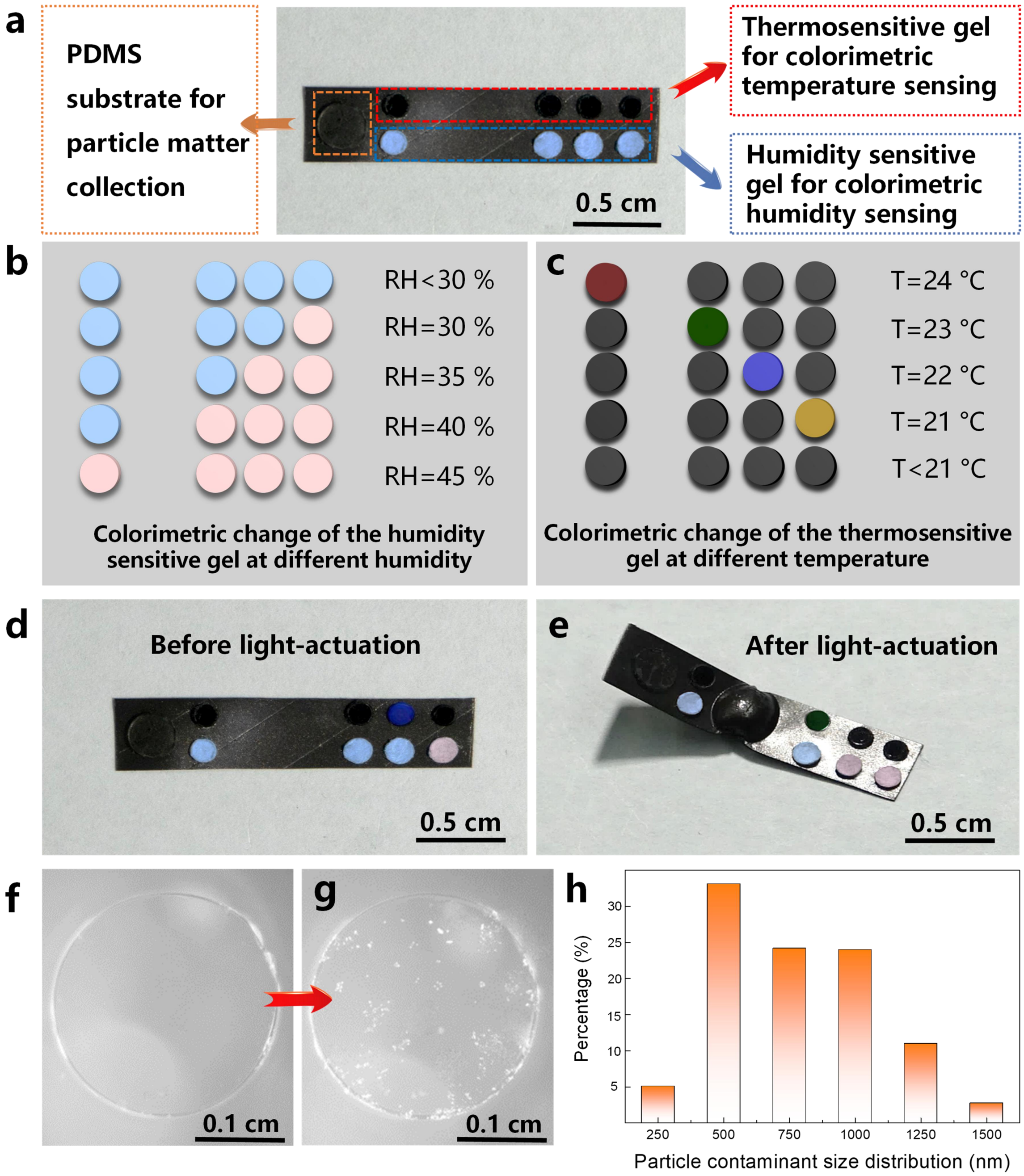

Supplementary Fig. 41. (a) CCD image showing the photoactuator integrated with a PDMS substrate, four thermosensitive gels and four humidity sensitive gels. (b) Colorimetric change of the humidity sensitive gel at different humidity. (c) Colorimetric change of the thermosensitive gel at different temperature. CCD images showing the photoactuator with surface-attached sensors and PDMS substrate (d) before and (e) after light actuation. Optical microscopic images of the surface-attached PDMS substrate (f) before release and (g) after landing (the release height and the wind speed are 1.5 m and 3 m/s, respectively). (h) Size distribution of the particulate pollutant.

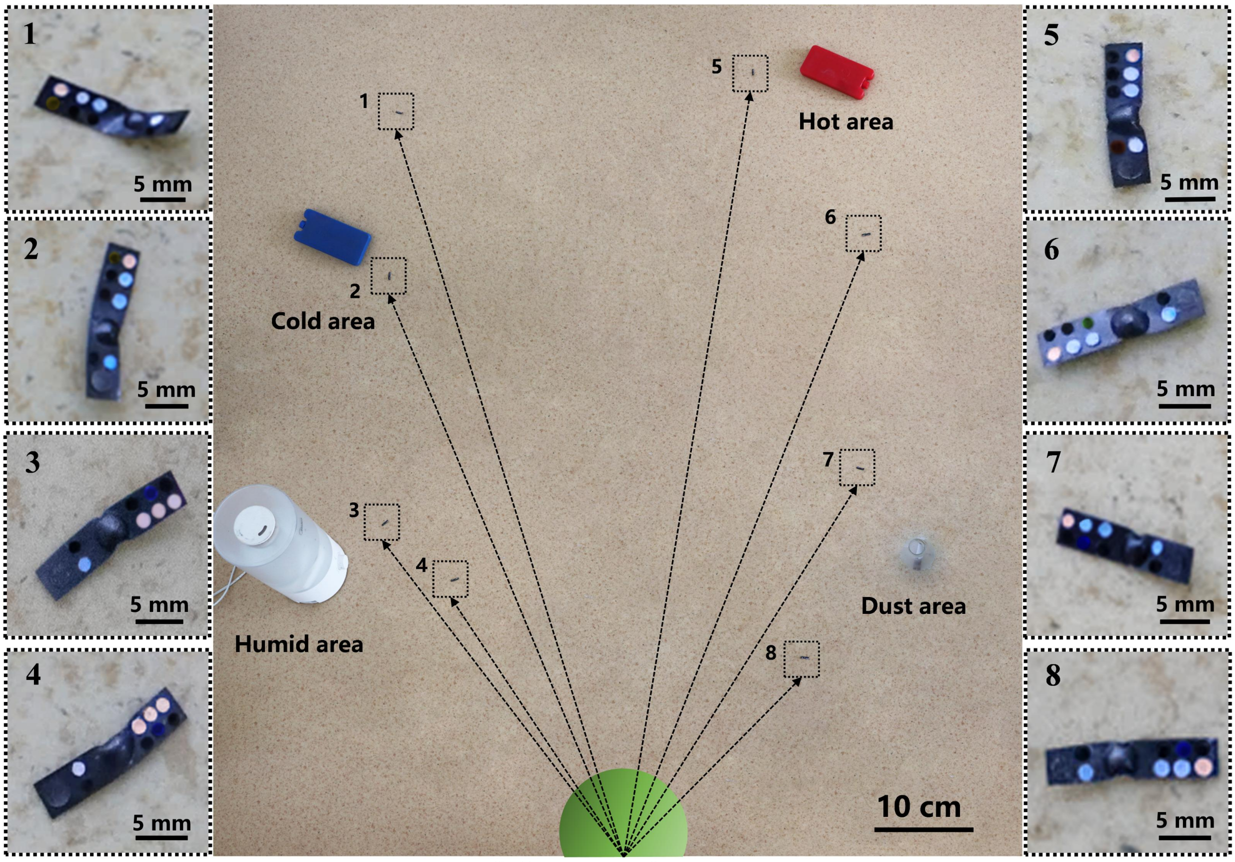

Supplementary Fig. 42. Digital image of the distributed photoactuators for collective environmental monitoring. The hot, cold, humid and dusty areas are all indicated. The corresponding enlarged images at different locations (from 1 to 8) are also shown. In order to demonstrate both clockwise and counterclockwise rotational films could be successfully deployed, we have released six films (i.e. film No. 1,2,3,5,7,8) with clockwise rotation and two films (i.e. film No. 4,6) with counterclockwise rotation (totally eight films). On the surface of the films, there are two rows of sensitive gels. The row consisting of humidity sensitive gels is placed closer to the jet propulsion position. The row consisting of thermosensitive gels is placed farther to the jet propulsion position. In addition, because of the large occupied area of the group containing the entire six sensitive gels (three humidity sensitive gels and three thermosensitive gels), they are placed to the right of the jet propulsion position in case of the closewise rotational flying and to the left of the jet propulsion position in the case of the counterclosewise roational flying in order to realize the success flight.

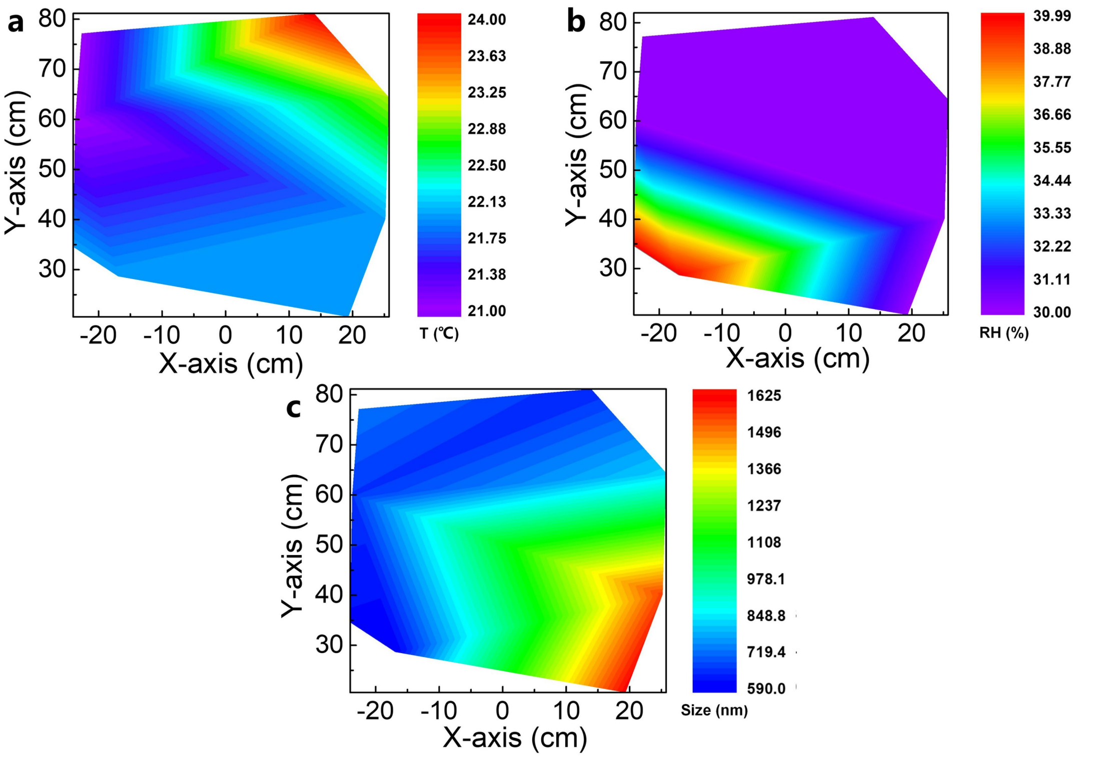

Supplementary Fig. 43. (a) Temperature distribution in the area shown in Supplementary Fig. 42 obtained based on the color analysis of the colorimetric temperature sensors on the landed photoactuators. (b) Humidity distribution in this area obtained based on the color analysis of the colorimetric humidity sensors on the landed photoactuators. (c) Size distribution of particulate pollution in this area which is obtained by analyzing the PDMS substrates on the surface of the landed photoactuators based on the dynamic light scattering (DLS) method.

Supplementary References

1. Shahsavan, H., Aghakhani, A., Zeng, H., Guo, Y., Davidson, Z. S., Priimagi, A. & Sitti, M. Bioinspired underwater locomotion of light-driven liquid crystal gels. *Proc. Natl. Acad. Sci. U. S. A.* **117**, 5125-5133 (2020).
2. Wang, T., Torres, D., Fernandez, F. E., Green, A. J., Wang, C. & Sepulveda, N. Increasing efficiency, speed, and responsivity of vanadium dioxide based photothermally driven actuators using single-wall carbon nanotube thin-films. *ACS Nano* **9**, 4371-4378 (2015).
3. Li, M., Wang, X., Dong, B. & Sitti, M. In-air fast response and high speed jumping and rolling of a light-driven hydrogel actuator. *Nat. Commun.* **11**, 3988 (2020).
4. Li, J., Zhang, R., Mou, L., Jung de Andrade, M., Hu, X., Yu, K., Sun, J., Jia, T., Dou, Y., Chen,

H., Fang, S., Qian, D. & Liu, Z. Photothermal bimorph actuators with in-built cooler for light mills, frequency switches, and soft robots. *Adv. Funct. Mater.* **29**, 1808995 (2019).

5. Wang, W., Liu, Y., Liu, Y., Han, B., Wang, H., Han, D., Wang, J., Zhang, Y. & Sun, H. Direct laser writing of superhydrophobic PDMS elastomers for controllable manipulation via marangoni effect. *Adv. Funct. Mater.* **27**, 1702946 (2017).
6. Maggi, C., Saglimbeni, F., Dipalo, M., De Angelis, F. & Di Leonardo, R. Micromotors with asymmetric shape that efficiently convert light into work by thermocapillary effects. *Nat. Commun.* **6**, 7855 (2015).
7. Kobayashi, M. & Abe, J. Optical motion control of maglev graphite. *J. Am. Chem. Soc.* **134**, 20593-20596 (2012).
