# Supplementary material for: Bioinspired rotary flight of light-driven composite films
Source: Nat Commun. 2023 Aug 21;14:5070. doi: 10.1038/s41467-023-40827-4 (PMC10442326; doi:10.1038/s41467-023-40827-4)
Supplement: Supplementary file 3 — Description of Additional Supplementary Files [file 41467_2023_40827_MOESM3_ESM.pdf]

Description of Additional Supplementary Files

Supplementary Movie 1. The rotary flight motion of the photoactuator.

Supplementary Movie 2. Deformation process indicating the formation of the 'airscrew'-like structure.

Supplementary Movie 3. Origination of the driving force, i.e., jetpropulsion.

Supplementary Movie 4. Light-driven rotation in the vacuum environment.

Supplementary Movie 5. Light-driven rotation with negative angle of attack.

Supplementary Movie 6. Lightcontrolled flight to the left rear.

Supplementary Movie 7. Lightcontrolled flight to the right rear.

Supplementary Movie 8. Mimic of the vine maple seed: synchronous rotaryfalling.

Supplementary Movie 9. Wind dispersal of the light-driven 'helicopter'.

Supplementary Movie 10. Photoactuator film without the 'airscrew'-like structure tumbles during the falling process.

Supplementary Movie 11. Lightdriven 'helicopter' flying across a trench.

Supplementary Movie 12. Lightdriven 'helicopter' flying over a barrier.
